# Development of Dried Blood Spot Proficiency Testing Materials for Newborn Screening of Lysosomal Diseases Using Recombinant Enzymes

**DOI:** 10.3390/ijns12020040

**Published:** 2026-06-09

**Authors:** Elya Courtney, Samantha L. Isenberg, Timothy Lim, C. Austin Pickens, Rachel Lee, Carla Cuthbert, Konstantinos Petritis

**Affiliations:** Division of Laboratory Sciences, National Center for Environmental Health, Centers for Disease Control and Prevention, Atlanta, GA 30341, USA; ecourtney2@cdc.gov (E.C.); sisenberg@cdc.gov (S.L.I.); thl5@cdc.gov (T.L.); ogh6@cdc.gov (C.A.P.); nmo1@cdc.gov (R.L.); ijz6@cdc.gov (C.C.)

**Keywords:** dried blood spot (DBS), recombinant enzymes, proficiency testing (PT), newborn screening (NBS), lysosomal diseases (LDs)

## Abstract

Lysosomal diseases (LDs, or Lysosomal Storage Disorders) have become increasingly visible in the newborn screening community, with the addition of mucopolysaccharidosis type II (MPS-II) into the Recommended Uniform Screening Panel in August 2022 and Infantile Krabbe disease in June 2024. As more LDs are expected to be considered for screening adoption, the ability to multiplex conditions and expand proficiency testing (PT) using quality control materials is essential. This study examines the use of recombinant enzymes to produce first-tier PT materials for mucopolysaccharidosis type I, MPS-II, Gaucher, Fabry, Krabbe, Pompe, and Niemann–Pick A/B (acid sphingomyelinase deficiency)—adding four disorders to the CDC’s Newborn Screening Quality Assurance Program (NSQAP) LD PT panel. Through an iterative process that included two prototype phases, two pilot phases, and external testing by up to 31 external laboratories, a new manufacturing process was developed for producing high-performing dried blood spot-based LD PT specimens. Materials were evaluated using several methods commonly employed by newborn screening laboratories, including tandem mass spectrometry with flow injection and liquid chromatography, digital microfluidics, and fluorometric assays. This novel process for producing LD PT materials offers several advantages over previous manufacturing methods that relied on immortalized cell lines from affected patients. Improved scalability, for example, has enabled NSQAP to expand LD PT enrollment internationally. Furthermore, the new process makes it easier to support future expansions of the LD screening panel. The updated specimens and expanded program were launched in January 2025.

## 1. Introduction

The Centers for Disease Control and Prevention’s (CDC) Newborn Screening Quality Assurance Program (NSQAP) provides first-tier lysosomal disease (LD, also known as Lysosomal Storage Disorder) proficiency testing (PT) materials to participating newborn screening (NBS) laboratories. This assists with quality assurance (QA) activities, minimizing incorrect results while supporting the implementation of screening for additional disorders. This program previously included mucopolysaccharidosis type I (MPS-I), Krabbe, and Pompe diseases [1] and was available to US laboratories. LD PT materials should include samples that are deficient in only one enzyme to flag as clinically significant for one condition, while also resulting within normal limits in clinical assessments for all other conditions included in that panel. This mimics results observed in specimens from newborns with LDs, where low enzymatic activity would trigger a preliminary positive result in newborn screening.

The burden of a positive screen is well established, and it is important to minimize false-positive screens to reduce these burdens on families and healthcare infrastructure [2]. False-negative screens can be devastating, sometimes resulting in delayed diagnosis, and can lead to permanent disability in the patient [3]. While neither are fully preventable, dried blood spot (DBS) PT materials are invaluable training and assessment tools for laboratories and are useful for evaluating assay performance, especially for rare conditions where residual disease specimens are difficult to acquire [4]. These contrived specimens could also be used in place of disease specimens for analytical method development and validation, as well as in blinded analyst evaluations.

**Figure 1:**
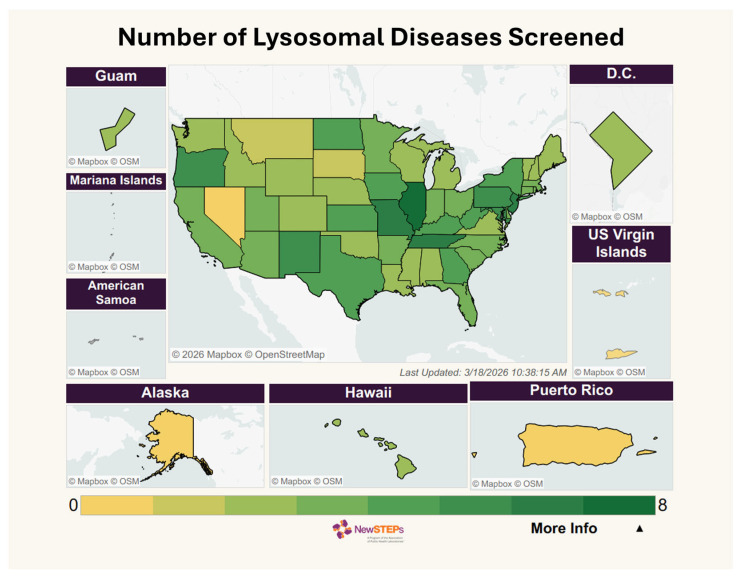
Heat map showing the number of lysosomal diseases (MPS I, MPS II, Gaucher, Fabry, Krabbe, Pompe, and Niemann–Pick A/B, or acid sphingomyelinase deficiency) screened by each U.S. state, based on data from the NewSTEPs database as of March 2026, provided by the Association of Public Health Laboratories and adapted with permission (2026, APHL). The eighth disease counted is Metachromatic Leukodystrophy, MLD.

In US states and territories, as of March 2026, 10 states screen for all LDs in the RUSP. In total, 50 states or territories screen for Pompe, 48 screen for MPS-I, 22 screen for MPS-II, and 19 screen for Infantile Krabbe disease (Figure 1). Eight states reported screening for additional LDs from those currently on the RUSP as part of the NewSTEPs program as of March 2026, with others currently validating assays for additional conditions [5]. As state newborn LD screening programs grow, expansion of the NSQAP LD PT program is an important step to support validation and adoption efforts. The recent additions of MPS-II and Infantile Krabbe disease to the RUSP are driving widespread efforts to expand NBS panels, making this updated program a timely resource provision.

Previous PT panels for LDs were produced using immortalized cell lines from affected patients [6]. Although these were fit for purpose in producing Pompe, Krabbe, and MPS-I PTs offered at the time, cell line-based expansion for future program needs has significant limitations. Already, recombinant alpha-L-iduronidase (IDUA) has been used to correct low activity in the GALC-deficient cell line that was used to create Krabbe PT specimens, and individual enzyme activities cannot be adjusted using the cell lines alone. Enrichments of cells are also not very consistent, are resource intensive, and are produced in-house, limiting production volume. Pilot work showed that additional supplementation of recombinant enzymes would be needed for a panel expansion using cell lines to manufacture MPS-II PTs. It would be difficult and time-consuming to find and grow appropriate and durable cell lines for a further expanded panel, as these cell lines must first be identified and then contracted for production-level growth. This process would need to be completed for every additional disorder. Recombinant enzymes are commercially available for many of the pathways associated with LDs, and these would be uniquely adjustable for desired enzymatic activity. Thus, using recombinant enzymes provides a more scalable and sustainable solution for PT material production by eliminating the need for identification, contracting, and expansion of disease-specific cell lines for each additional disorder, which would otherwise be required for continued panel expansion.

## 2. Materials and Methods

Preliminary work included performing enzyme titrations in blood, attempting direct enrichment from enzyme buffers into blood base pools. Initial base pools comprised doubly leukodepleted red blood cells (ZenBio, Inc, now BioIVT, Hicksville, NY, USA), hematocrit (hct) adjusted to 50% using heat-treated charcoal-stripped human serum (Seracare: SeraCon™ II CD Hormone Depleted Negative Diluent; 1800-0006, Milford, MA, USA). Serum was heat-treated at 56 °C for 4 h in a heated water bath while stirring. Initial titrations informed a linear titration in blood enriched with 7 recombinant enzymes, as shown in Supplemental Table S1 (ST), purchased from BioTechne (Minneapolis, MN, USA). This activity was reviewed by CDC, which deemed that the research did not involve human subjects and was consistent with applicable federal law and CDC policy.

GALC and I2S activities were notably lower than other species (Supplement Figure S1) in the high titration pool. Computations for made-to-order GALC and I2S enzyme amounts were then performed to ensure sufficient activities approximating NBS medians in future PT pools, since initial amounts used were in the standard catalog. These specimens were made by enriching recombinant enzymes in buffer directly from the vial into a pool of doubly leukodepleted blood, adjusted to 50% hct with heat-treated charcoal-stripped human serum. Recombinant titration DBSs were sent to external laboratories for assessment and informed calculations for enrichment processes and amounts for the PT specimens. Enrichments for the first prototype PTs were experimentally determined, since only minimum guaranteed enzyme activity per unit was provided for each species on the certificates of analysis (COA).

A secondary experiment to determine blood-processing methods was performed testing triply leukodepleted blood and heat-treating conditions to lower enzymatic activities in the base pool, and double leukodepletion with heat treatment of the hct-adjusted base pool for 90 min at 53 °C was found to be sufficient. Critically, the base pool should be hct adjusted to 50% using only charcoal-stripped human plasma, which was itself already heat-treated. This is different than previously published methods for preparing enzyme-depleted base pools with heat treatment protocols, as we found that using saline in the hct adjustment process prevented sufficient reduction in I2S enzyme activity [7].

**Figure 2:**
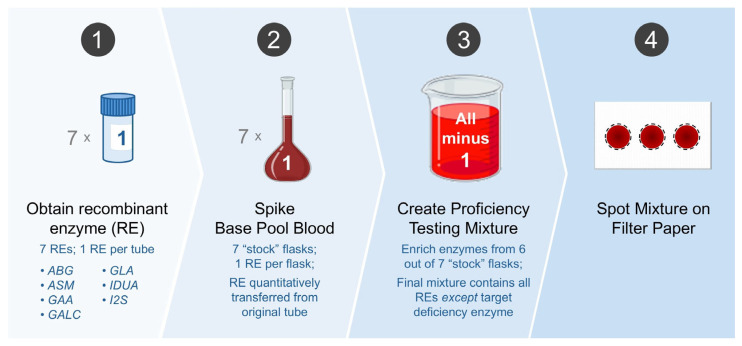
Final production workflow for recombinant enzyme-enriched LD PT specimens, shown in four stages.

To achieve consistent enrichments of the recombinant enzymes for the second prototype and all subsequent productions, these enzymes were quantitatively transferred from their vials using the heat-treated base pool blood into labeled volumetric flasks for prototype, pilot, and formal productions (Figure 2). Enrichments were calculated based on recombinant enzyme concentrations in the volumetric flasks as determined by masses on the COAs. After the second prototype material results were analyzed, enrichments for each enzyme were adjusted before pilot production to aim closer to the NBS median activity.

The current panels include PT specimens for conditions in Table 1. Using the workflow in Figure 2, base pool blood was used to quantitatively transfer each enzyme into small volumetric flasks. These stock solutions of recombinant enzyme were then used to enrich larger PT pools, enriching all but the target deficiency for assessment.

All specimens were assessed internally and externally with kits and lab-developed methods, using flow injection tandem mass spectrometry (FIA-MS/MS), liquid chromatography tandem mass spectrometry (LC-MS/MS), and fluorometric methods during development and pilot stages to ensure material fitness and provide process feedback [6,8,9]. Internal development work and characterizations prior to initiating pilot studies and program deployment were completed using FIA-MS/MS 6-Plex methods and LC-MS/MS 7-plex and 1-plex (I2S) methods, using cold-induced phase separation for sample clean-up [10]. I2S external results, cutoffs, and medians were normalized to internal methods for development purposes using CDC LD QC values reported with the prototype specimen data and averaged for comparison. Average values from reported results, cutoffs, and medians from states reporting data acquired by MS/MS analysis were used for the other 6 enzymes. This provided additional confidence that enrichments would approximate reported NBS medians and avoid borderline values across multiple sample preparation methods and analysis platforms prior to finalizing the production enrichment protocol. Final preparations and characterizations were performed using the NeoLSD™ MSMS kit (Revvity, Waltham, MA, USA) and an in-house 1-plex LC-MS/MS (I2S) laboratory-developed test on a QSight 225MD system (Revvity, Waltham, MA, USA).

## 3. Results

### 3.1. Prototype Studies and Development

Three external laboratories assessed materials for the first prototype study. The clinical assessments of the ABG, ASM, I2S, and GAA enzymes were correct. GLA, GALC, and IDUA PT specimens (where these enzymes should be uniquely deficient in the panel) were not sufficiently depleted in the base pool, showing incorrect clinical assessments for two labs for GLA (66% false negative) and one lab for GALC and IDUA (33% false negative). After developing the quantitative transfer process and changing the depletion protocol for the base pool, an additional prototype production occurred to adjust enrichments. The second prototype round was assessed by six laboratories, with clinical assessments reported at 100% accuracy for all specimens. The target enrichments result in enzyme activities that approximate newborn screening medians for each species, as reported by state public health laboratories during the development process. Lot-based variations in activity were assessed concurrently with the materials pilot studies, building safeguards into the production process to avoid creating borderline specimens.

### 3.2. Pilot Studies

Formal pilot studies were conducted through the NSQAP Participant Portal in May and September 2024 by sending blinded panels of five DBS PT specimens to participants enrolled in the current LD PT program. These panels covered seven conditions, with targeted deficiencies as listed in Table 1. Enzyme activities, clinical assessments, cutoffs, and analysis methods were reported through the portal by participating laboratories. NBS medians were reported during prototype studies. Data for NBS cutoffs and medians (stratified by analysis method) were utilized to further improve the enrichment of the production-grade materials to better match the median enzymatic activity levels observed in newborns. Laboratories reported clinical assessments with 100% accuracy for the LD PT Pilot 1 and 99% accuracy for LD PT Pilot 2, as shown in Tables S3 and S4.

**Figure 3:**
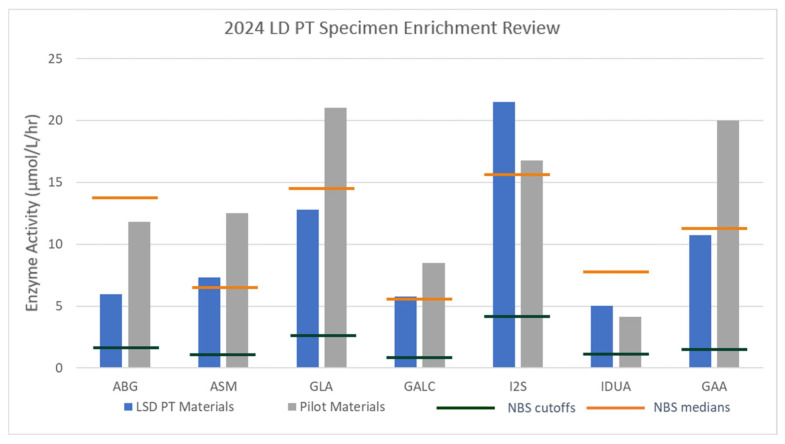
FIA-MS/MS (I2S: LC-MS/MS) characterization data for Pilot 1 and production-grade LD PT specimens enriched with recombinant enzymes. Average harmonized NBS medians and cutoffs for each analyte across study participants are also shown. I2S values were normalized to reported CDC QC data.

Figure 3 shows enrichment improvements from pilot to production-grade specimens using the FIA-MS/MS data reported by external laboratories, as compared to reported medians and cutoffs. Data were stratified by analysis method for evaluation purposes during development, since there were notable differences in values between mass spectrometry and fluorometric methods [11]. MS/MS data were used to inform enrichment adjustments from pilot to 2024 program specimens.

### 3.3. Program Deployment

The expanded NSQAP LD PT program was deployed in January 2025 to all domestic programs previously enrolled for LD PT, and an additional 40 international laboratories which then enrolled for participation. In total, 71 laboratories reported clinical assessments, enzymatic activities, cutoffs, and methods used to determine the activity of each enzyme; 98% of results were considered acceptable/correct.

In PT data from Q1 2025 (Table 2), more than half of the incorrect assessments were related to sample mishandling or data reporting errors. For the Fabry-deficient specimen, activities were below all reported cutoffs with an incorrect clinical assessment by one laboratory. Another laboratory reported an incidental switch of results for MPS-I and MPS-II. For the IDUA-deficient specimen, one laboratory submitted an incorrect clinical assessment for a result with activity below the reported cutoff. Two laboratories reported ASM activities below their cutoffs on the normal specimen, and one reported IDUA activity slightly above their cutoff in the MPS-I PT.

## 4. Discussion

Surveillance of enzymatic activities in PT specimens and lot-to-lot variations in recombinant enzyme activities will inform future adjustments and enrichment buffer zones, ensuring values are not borderline to cutoffs for any enriched species. A clear example of recombinant enzyme lot-to-lot variation is the difference in ABG activities between pilot and production-grade specimens, as recombinant enzyme levels were not adjusted between productions. ABG and IDUA enrichments will be increased in the next production to better match reported NBS median activities. Continued assessment of these factors will be needed as enzymes are added to the panel to produce PT specimens for additional conditions. As more LDs are routinely screened using diverse methods and more data become available, median and cutoff assessments should continue using the normalization procedure to ensure materials remain fit for purpose. Normalization of the reported PT data to reported QC data will continue for ongoing enrichment assessments, as there are many factors that can influence calculated enzymatic activities and recoveries.

One of the concerns when using recombinant enzymes to create PT specimens was the high concentrations of additives included in their formulations that are not friendly to mass spectrometry analysis. These included glycerol up to 20% by volume, Tris up to 25 mM, and sodium chloride up to 400 mM. We encountered ion-suppression effects during mass spectrometry analysis of high recombinant enzyme enrichments during the titration experiments. The fluorometric methods were not affected by the recombinant enzyme buffer additives in the high-level titration, indicating that the issues are isolated to FIA and LC-MS/MS based methods and supporting the hypothesis that suppressive effects are, in part, due to ionization issues in the presence of excess salts and glycerol in the DBS matrix. We do not anticipate ion suppression from buffer components in recombinant enzyme formulations to be a near-term issue when adding enzymes to the LD PT panel. In contrast, buffer concentrations in production pools are substantially lower, as enrichments are designed to approximate newborn screening median enzyme activities. The total volume of recombinant enzyme buffer added to any production pool is less than 25% of that used under conditions where ion suppression was observed. As additional LDs are incorporated into screening panels and more recombinant enzymes are introduced, potential ion suppression from buffer additives will continue to be evaluated.

## 5. Conclusions

The novel manufacturing protocol described herein is fit for purpose to expand the NSQAP first-tier LD PT program to include seven conditions, as specimens were assessed successfully using currently utilized mass spectrometry and fluorometric methods. Fabry, MPS-II, Gaucher, and Niemann–Pick A/B PTs are included for the first time, and this shows promise for expansion as additional LDs are considered for NBS. These PT specimens support laboratories in their compliance and quality efforts, eliminating the need for residual DBS exchange programs to meet proficiency testing requirements in regions that screen for these disorders with MS/MS and fluorometric methods. Successful pilot studies and initial deployment of the expanded program have also enabled international laboratories that participate in NSQAP to enroll in the new LD PT program, with 40 additional laboratories enrolled in 2025. Analysis of reported PT results will continue to inform improvements for future productions.

## Figures and Tables

**Table 1:** Enzyme names, acronyms, and the LDs associated with deficiencies of these enzymes included in the initial expansion of the first-tier LD PT panel.

Enzyme	Acronym	Associated Disorder
α—galactosidase A	GLA	Fabry
Acid α—glucosidase	GAA	Pompe
Acid β—glucosidase	ABG (GBA)	Gaucher
Acid sphingomyelinase	ASM	Niemann–Pick A/B
α—L—iduronidase	IDUA	MPS-I
Galactosylceramidase	GALC	Krabbe
Iduronate-2-sulfatase	I2S (IDS)	MPS-II

Each PT pool is enriched with every recombinant enzyme listed here, except for the target disorder. Example: Pompe PT specimens are enriched with each recombinant enzyme except GAA.

**Table 2:** Numbers reflect the number of laboratories that reported values and assessments for a given enzyme by sample in Q1 2025. The proportion of correct clinical assessments is shown against the total number of labs reporting for a given specimen and analyte. Highlighted cells show deficient enzyme for a given PT specimen.

	*Normal*	*Krabbe*	*Fabry*	*MPS-I*	*MPS-II*
***ABG***	32/32	32/32	32/32	32/32	32/32
***ASM***	23/25	25/25	25/25	25/25	25/25
***GAA***	63/63	63/63	63/63	63/63	63/63
***GALC***	34/34	34/34	34/34	34/34	34/34
***GLA***	35/35	35/35	34/35	35/35	35/35
***I2S***	25/25	25/25	25/25	25/25	24/25
***IDUA***	64/64	64/64	64/64	62/64	64/64

## Data Availability

The data relevant to this study are presented in this article and in the Supplemental Information. Requests for additional data can be directed to the corresponding author.

## Supplementary Materials

The following supporting information can be downloaded at: https://www.mdpi.com/article/10.3390/ijns12020040/s1. Figure S1: Recombinant enzyme linear titration results; Table S1: Recombinant enzyme product information; Table S2: Recombinant enzyme titration enrichments; Table S3: Pilot 1 results; Table S4: Pilot 2 results.
