# Supplementary material for: Development of Dried Blood Spot Proficiency Testing Materials for Newborn Screening of Lysosomal Diseases Using Recombinant Enzymes
Source: Int J Neonatal Screen. 2026 Jun 9;12(2):40. doi: 10.3390/ijns12020040 (PMC13299422; doi:10.3390/ijns12020040)
Supplement: Supplementary file 1 [file IJNS-12-00040-s001.zip › IJNS-4282539-supplementary.pdf]

Product Name	Bio-Techne Product Number
Recombinant Human galactosylceramidase protein	7310-GH-MTO (50µg)
Recombinant Human SMPD1	5438-PD-010
Recombinant Human glucosylceramidase/GBA	7410-GHB-020
Recombinant human alpha-L-iduronidase	4119-GH-010
Recombinant Human alpha-glucosidase	8329-GH-025
Recombinant Human alpha-galactosidase-A	6146-GH-020
Recombinant Human Iduronate-2-sulfatase protein	2449-SU-MTO (100µg)

Supplemental Table 1: Product names and product numbers for the recombinant enzymes used to enrich the contrived PT specimens. Recombinant enzymes were purchased from R&D Systems, a Bio-Techne company (Minneapolis, MN, USA). MTO designation in product numbers indicates that the amount of enzyme for those species was more than a stock vial, and the amounts purchased for the Q12025 production are in parenthesis.

Enzyme	ABG	ASM	GAA	GALC	GLA	IDUA	I2S
Enrichment (µg)	20	10	25	5	20	10	20
Buffer volume added (µL)	25	25	25	250	25	25	250

Supplemental Table 2: Amounts of each enzyme presumed enriched in the high pool (volume: 25mL) for linear titrations, along with the volume of enzyme buffers added to the blood pool (entire contents of each vial).

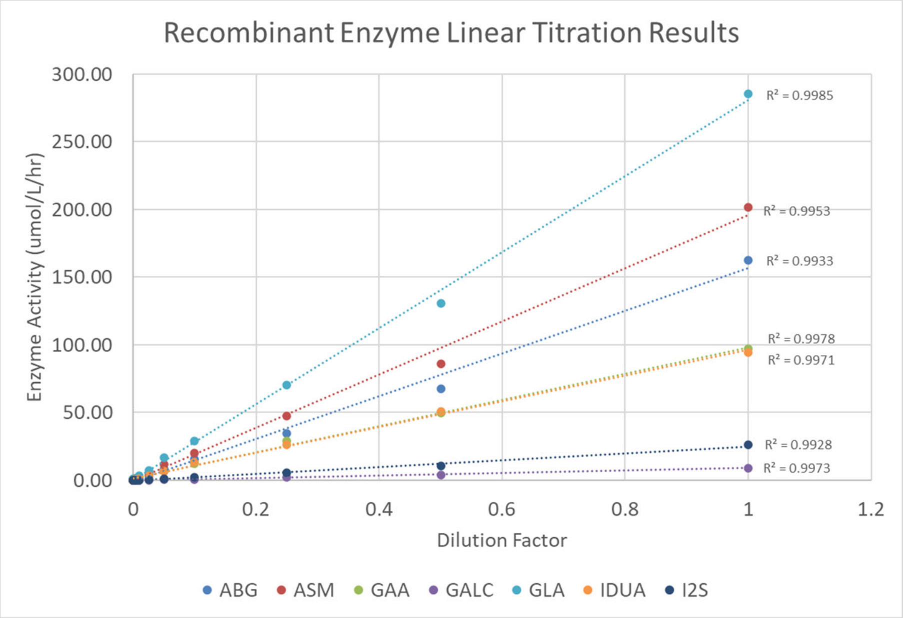

Supplemental Figure 1: Enzyme activities achieved for each analyte shown against the dilution factor for the enriched blood pools created during recombinant enzyme linear titrations. Enrichments in the high pool are as described in ST2.

	Gaucher	Niemann-Pick A/B	Pompe	Krabbe	MPS-II
ABG	7/7	7/7	7/7	7/7	7/7
ASM	4/4	4/4	4/4	4/4	4/4
GAA	21/21	21/21	21/21	21/21	21/21
GALC	7/7	7/7	7/7	7/7	7/7
GLA	8/8	8/8	8/8	8/8	8/8
I2S	8/8	8/8	8/8	8/8	8/8
IDUA	20/20	20/20	20/20	20/20	20/20

Supplemental Table 3: The proportion of correct clinical assessments is shown against the total number of labs reporting for a given specimen and analyte for the first Pilot event in May 2024. Highlighted cells show deficient enzyme for a given PT specimen. All clinical assessments were correct (22 laboratories reported results).

	Normal	Niemann-Pick A/B	Fabry	MPS-I	MPS-II
**ABG**	11/11	11/11	11/11	11/11	11/11
**ASM**	8/8	8/8	8/8	8/8	8/8
**GAA**	31/31	31/31	31/31	31/31	31/31
**GALC**	13/13	13/13	13/13	13/13	13/13
**GLA**	14/14	14/14	14/14	14/14	14/14
**I2S**	14/14	14/14	14/14	14/14	14/14
**IDUA**	29/30	29/30	29/30	30/30	29/30

Supplemental Table 4: The proportion of correct clinical assessments is shown against the total number of labs reporting for a given specimen and analyte for the second Pilot event in September 2024. Highlighted cells show deficient enzyme for a given PT specimen. 99% of the clinical assessments were correct (31 laboratories reported results).
